# Integrative analysis associates monocytes with insufficient erythropoiesis during acute *Plasmodium cynomolgi* malaria in rhesus macaques

**DOI:** 10.1186/s12936-017-2029-z

**Published:** 2017-09-22

**Authors:** Yan Tang, Chester J. Joyner, Monica Cabrera-Mora, Celia L. Saney, Stacey A. Lapp, Mustafa V. Nural, Suman B. Pakala, Jeremy D. DeBarry, Stephanie Soderberg, the MaHPIC Consortium, Jessica C. Kissinger, Tracey J. Lamb, Mary R. Galinski, Mark P. Styczynski

**Affiliations:** 1 School of Chemical & Biomolecular Engineering, Georgia Institute of Technology, Atlanta, GA USA; 2 Malaria Host–Pathogen Interaction Center, Emory Vaccine Center, Yerkes National Primate Research Center, Emory University, Atlanta, GA USA; 3 Institute of Bioinformatics, University of Georgia, Athens, GA USA; 4 Department of Genetics, University of Georgia, Athens, GA USA; 5 Center for Tropical and Emerging Global Diseases, University of Georgia, Athens, GA USA; 6 Department of Computer Science, University of Georgia, Athens, GA USA; 7 Department of Pathology, University of Utah, Salt Lake City, UT USA; 8 Division of Infectious Diseases, Department of Medicine, Emory University, Atlanta, GA USA

**Keywords:** *Plasmodium cynomolgi*, GATA1, Nonhuman primates, Relapse, *Plasmodium vivax*, Bone marrow, Erythropoiesis, Transcriptomics, Systems biology, Immune response

## Abstract

**Background:**

Mild to severe anaemia is a common complication of malaria that is caused in part by insufficient erythropoiesis in the bone marrow. This study used systems biology to evaluate the transcriptional and alterations in cell populations in the bone marrow during *Plasmodium cynomolgi* infection of rhesus macaques (a model of *Plasmodium vivax* malaria) that may affect erythropoiesis.

**Results:**

An appropriate erythropoietic response did not occur to compensate for anaemia during acute cynomolgi malaria despite an increase in erythropoietin levels. During this period, there were significant perturbations in the bone marrow transcriptome. In contrast, relapses did not induce anaemia and minimal changes in the bone marrow transcriptome were detected. The differentially expressed genes during acute infection were primarily related to ongoing inflammatory responses with significant contributions from Type I and Type II Interferon transcriptional signatures. These were associated with increased frequency of intermediate and non-classical monocytes. Recruitment and/or expansion of these populations was correlated with a decrease in the erythroid progenitor population during acute infection, suggesting that monocyte-associated inflammation may have contributed to anaemia. The decrease in erythroid progenitors was associated with downregulation of genes regulated by GATA1 and GATA2, two master regulators of erythropoiesis, providing a potential molecular basis for these findings.

**Conclusions:**

These data suggest the possibility that malarial anaemia may be driven by monocyte-associated disruption of GATA1/GATA2 function in erythroid progenitors resulting in insufficient erythropoiesis during acute infection.

**Electronic supplementary material:**

The online version of this article (doi:10.1186/s12936-017-2029-z) contains supplementary material, which is available to authorized users.

## Background

*Plasmodium vivax* infections cause substantial morbidity with an estimated 8.5 million infections each year and can result in severe disease [1]. Anaemia may occur during primary or relapse infections caused by *P. vivax* and may be due, in part, to bone marrow (BM) dysfunction as evidenced by post-mortem examinations of malaria patients [2]. This dysfunction leads to dyserythropoiesis and insufficient erythropoietic output to compensate for the loss of red blood cells (RBCs) from parasitism as well as immune-mediated removal of uninfected RBCs [3–6]. The underlying mechanisms and characteristics of BM dysfunction in humans remain poorly understood [2, 7, 8] due to the difficulties and ethical restrictions in obtaining longitudinal bone marrow samples from patients. Animal models can overcome this obstacle and be used to investigate mechanisms related to the development and recovery of BM dysfunction during malaria.

Nonhuman primate (NHP) macaque models are optimal for anaemia studies in that they show haematopoietic responses and erythropoietic processes that are very similar to those of humans, and BM aspirates can be collected at multiple time points during longitudinal malaria studies [9, 10]. Although rodent models of malaria demonstrate overarching similarities in the development of anaemia, differences in the haematopoietic responses between rodents and humans impose limitations on the extrapolation of some conclusions [11, 12]. For example, there are notable differences in the transcriptional programmes that underlie erythropoiesis in mice compared to humans and different physiological mechanisms to deal with anaemia [13].

The *Macaca mulatta*–*Plasmodium cynomolgi* model system recapitulates critical aspects of *P. vivax* infection in patients, including BM dysfunction and insufficient erythropoiesis during acute infections [10, 14]. Importantly, *P. cynomolgi* also produces hypnozoites in the liver of rhesus macaques, enabling the study of relapse infections that occur with *P. vivax* in patients [9, 10, 15]. There has been extensive study of malaria-induced anaemia in *Plasmodium falciparum*, in particular in the study of severe malarial anaemia. Comparatively, much less is known about the pathogenesis of anaemia during *P. vivax* infection, even though it is increasingly recognized that *P. vivax* also causes substantial anaemia [16]. In fact, *P. vivax* causes a greater removal of uninfected red blood cells than *P. falciparum* [4]. Furthermore, the effect of relapse infections on the bone marrow in comparison to an initial infection has not been thoroughly characterized even though relapses are speculated to potentially drive anaemia in areas of high endemicity with frequent relapses [17].

Here, a malaria systems biology study is presented that used BM samples from a cohort of *Macaca mulatta* infected for about 100 days with *P. cynomolgi* M/B strain [9] as a model of vivax malaria. The main goal was to perform integrative analyses and identify potentially dysfunctional BM mechanisms that may contribute to anaemia during acute vivax malaria and relapses. Multiple data types were generated, analysed, and integrated (i.e. transcriptome, immune profiling, and clinical data). In this paper, the analysis of the bone marrow transcriptome during the infection is presented, focusing on large-scale changes via pathway analysis and highlighting the inflammation in the marrow during acute infection. Next, the cell types that may be responsible for these changes in the marrow are explored, and the upregulation of immune pathways identified via transcriptional profiling is validated by measuring systemic cytokine levels. Finally, the consequence of these changes on erythroid progenitors is explored, with evidence suggesting the possibility that the function of GATA1/GATA2, which are known master regulators of erythroid differentiation, may be disrupted. Collectively, this is the first systems biology study using NHP models of *P. vivax* infection to study BM dysfunction and anaemia.

## Methods

### Animals

Samples from a cohort of male rhesus macaques (*Macaca mulatta*) born and raised at the Yerkes National Primate Research Center (YNPRC), an AAALAC international certified institution, were used in this study. All male animals were used to avoid anaemia related to the female menstrual cycle, which could confound interpretation of results. The animals were socially housed in pairs during the experiment, and all housing was in compliance with the Animal Welfare Act and Regulations, as well as the Guide for the Care and Use of Laboratory Animals. Details related to the animal’s daily care have been reported in Joyner et al. [9].

### Experimental design

Bone marrow aspirates collected from four *M. mulatta* prior to infection and during *P. cynomolgi* M/B strain infections initiated by sporozoite inoculation were analysed. The experimental design and clinical outcomes were described and analysed previously [9]. Clinical parameters are discussed in this manuscript as relevant.

### BM collection and processing

**Fig. 1:**
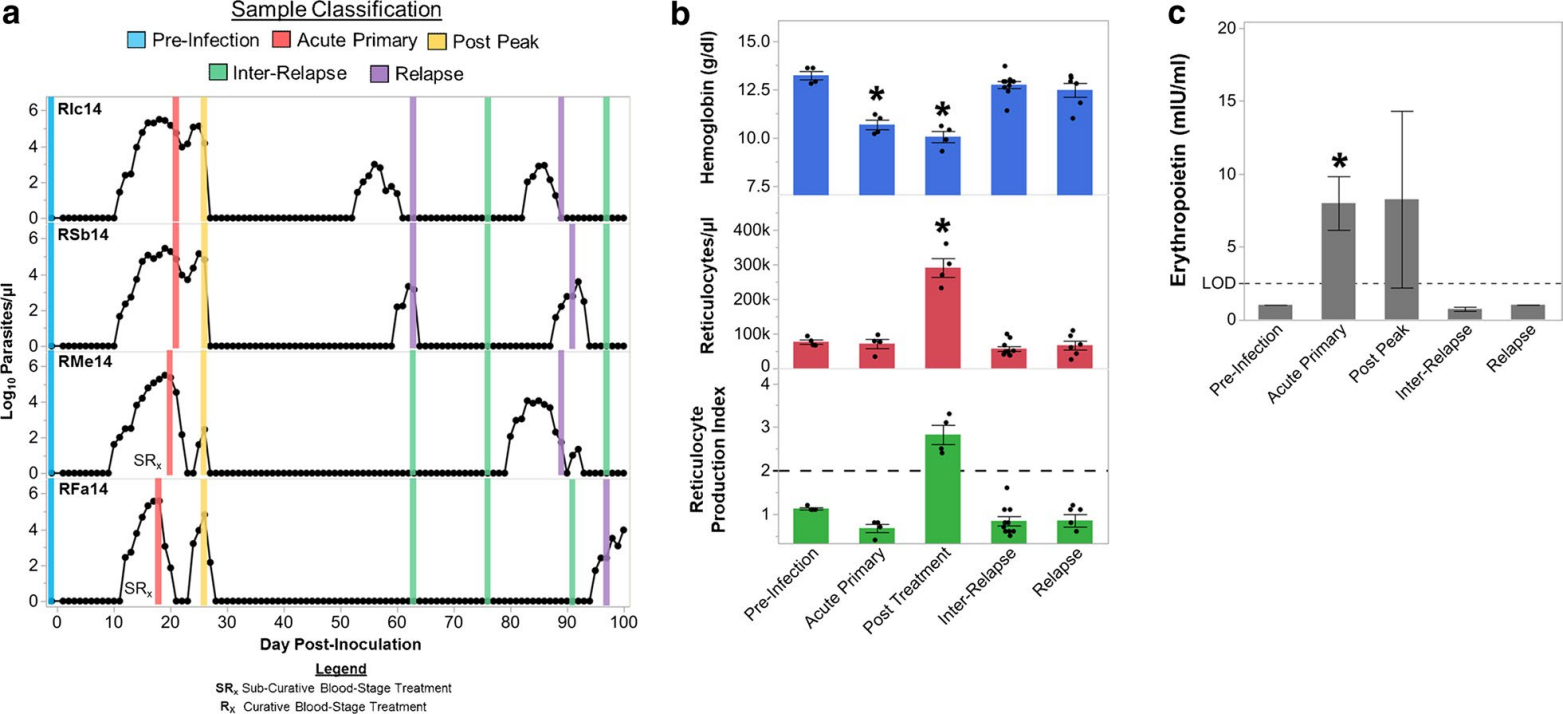
The bone marrow does not compensate for anemia during acute cynomolgi malaria despite increased EPO levels. **a** Parasitaemia kinetics for the four animals are shown. Bone marrow sample collection times in relation to parasite kinetics are indicated by a vertical bar; the colour of the bar indicates the infection point classification of the sample. **b** Hemoglobin levels, peripheral reticulocyte numbers, and the reticulocyte production index for each infection stage are shown. Statistical significance relative to pre-infection was assessed where relevant using a linear mixed-model with Tukey–Kramer post hoc analysis. **c** Mean erythropoietin levels during each infection stage are indicated. Dashed line indicates the limit of detection of the assay. Statistical significance was assessed using a paired t test relative to pre-infection levels. Error bars indicate standard error for **b** and **c**. Asterisk indicates p < 0.05

Seven BM aspirate specimens were collected in EDTA from each animal in the course of the 100-day experiment at pre-defined time points for simultaneous flow cytometry and RNA-Seq analysis as indicated in Fig. 1a. Flow cytometry samples were processed as described below. For RNA-Seq analysis, BM mononuclear cells (MNCs) were isolated via Lymphoprep (Stem Cell Technologies) following the manufacturer’s protocol. BM MNCs were immediately placed into RLT buffer (Qiagen) after isolation, vortexed, and stored at −80 °C for RNA-Seq analysis.

### Library preparation for RNA-Seq

RNA was extracted from BM MNCs using a Qiagen RNeasy Mini-Plus kit according to the manufacturer’s instruction. After extraction, the quality of each sample was evaluated with a Bioanalyzer. An RNA Integrity Number [18] greater than eight, signifying good RNA quality, was recorded for all samples prior to library preparation. Approximately 1 μg of total RNA per sample type was reverse transcribed into double-stranded cDNA, with Illumina TruSeq Stranded mRNA Sample Prep kits used to generate strand-specific libraries. For quality control, total RNA for each library contained approximately 1% spike-in RNAs of known concentration and GC proportions (ERCC Spike-In Control, Life Technologies) [19]. Sequencing was done on an Illumina HiSeq 2000 at the YNPRC Genomics Core. Each library averaged approximately 50 million 100 bp paired-end reads.

### Gene expression quantification

A whole-transcriptome profile of BM MNCs was generated by RNA-Seq, and over 50 million reads per sample were aligned jointly to concatenated host and parasite genomes (i.e. MacaM assembly, Version 4.0, GenBank accession number PRJNA214746 ID: 214746 and *P. cynomolgi* B/M strain PlasmoDB release-9.3) using Tophat2 with default parameters [20, 21]. Reads mapping to multiple genomic locations were excluded from analysis to ensure high-confidence mapping. Gene expression (read counts) were inferred at the level of annotated genes using HTSeq v0.5.4 [22]. Data reliability was assessed by several steps of quality control: linear correlation of spike-in control abundance with known concentration; confirmation of strand-specificity of controls as 99.9%; and confirmation of absence of 3′ bias in the controls with RSeqC software [23]. Gene expression was normalized to library size with the R package DESeq (version1.10.1; [24]), used with default parameters.

### RNA-Seq data processing

For macaque RNA-Seq, features with an FPKM value (fragments per kilobase of transcript per million mapped reads) below 5 were excluded from analysis. Gene expression data were log2 transformed. A large variance in gene expression was observed among animals (Additional file 1B); supervised normalization of microarrays (SNM) [25] was used to remove the variance associated with this animal effect. The full SNM model included longitudinal time point as the biological effect and animal as an adjustable effect. SNM-transformed RNA-Seq data was used for all analysis except for the initial hierarchical clustering analysis (Additional file 1A). All expression data were z-score normalized before integrative analysis.

### Differential expression analysis (DEA)

DEA was performed using analysis of variance in JMP Genomics. Differences in gene expression levels across all genes among time points were tested. Benjamini–Hochberg false discovery rates (FDR) [26] were used for multiple hypothesis correction, with FDR ≤ 0.05 used as the significance threshold.

### Weighted gene co-expression network analysis

Genes identified as differentially expressed during the acute infection were used for WGCNA with default parameters. The soft thresholding power parameter of WGCNA was set to six to obtain networks of approximately scale–free topology, as is standard practice, and the eigengene of each coexpression gene module was used to study associations with immunological traits.

### Flow cytometry

Two-hundred microlitres of BM aspirate to be used for flow cytometry was collected at the same time as RNA-Seq specimens. Bone marrow was initially washed with 2 mL of wash buffer [PBS + 2% Fetal Bovine Serum (FBS)] by centrifugation at 400×*g* for 5 min. After centrifugation, the supernatant was aspirated and discarded. The remaining cell pellet was suspended in PBS to the original volume followed by staining with antibodies as indicated in Table 1. The cells were stained with the antibody cocktails for 30 min at room temperature in the dark. After staining, the sample was placed into 2 mL of BD FACS Lysing solution (BD Biosciences), incubated for 10 min in the dark at room temperature, and the remaining cells pelleted by centrifugation at 400×*g* for 5 min. The cells were then washed in 3 mL of wash buffer by centrifugation at 400×*g* for 5 min. The supernatant was discarded and cells resuspended in a 2% paraformaldehyde and PBS solution (v/v) and analysed using a BD LSR-II flow cytometer within 24 h using a standardized acquisition template. Daily calibration using BD CST beads was performed to ensure direct comparability of each acquisition.

**Table 1:** Flow cytometry panel for determining the frequency of immune and erythroid cells in macaque bone marrow aspirate

Name	Fluorochrome	Clone
CD3	PerCp-Cy5.5	SP34-2
CD45	FITC	DO58-1283
CD41a	PE	HIP8
CD71	APC	LOl.1
CD11b	PE-Cy7	ICRF44
CD34	PE-CF594	563
CD44	APC-H7	G44.26
CD16	ALEXAFLUOR 700	3G8
CD14	PB	M5E2
CD20	V-500	L27

### Fluorescence-activated cell sorting (FACS)

Bone marrow MNCs were isolated as described above. Residual RBCs were lysed with 1–2 mL of ACK Lysing solution (Life Technologies) followed by washing with excess sterile PBS. BM MNCs were then enumerated using a Countess II cell counter (Life Technologies) and two million BM MNCs were stained with the fluorescently conjugated antibodies in Table 2 as described above followed by re-suspension in incomplete RPMI 1640 without FBS and phenol red for FACS. Populations of interest were sorted under optimal conditions using a FACS Aria II cell sorter (Beckman). After sorting, the purity of each population was confirmed to be ≥95% and populations were adhered to slides using a Cytospin followed by staining using Wright–Giemsa to determine the composition of the target populations.

**Table 2:** Cell sorting panel for erythroid progenitor populations in rhesus macaque bone marrow

Name	Fluorochrome	Clone
CD45	FITC	DO58-1283
CD41a	PE	HIP8
CD71	APC	LOl.1
CD34	PE-CF594	563

### Multiplex cytokine assay

A custom-made multiplex cytokine assay was designed and purchased from Affymetrix. The manufacturer’s suggested protocol was followed using plasma collected from blood specimens obtained on EDTA. Data was analysed using the ProcartaPlex Software.

### Erythropoietin ELISA

Erythropoietin levels were determined by using a Quantikine IVD ELISA assay for human erythropoietin using the manufacturer’s suggested protocol. All samples were randomized prior to performing the ELISA.

### Other statistical analysis

Limma was used for assessing significant changes in cytokine concentrations and cell population between infection points [27, 28]. The Benjamini–Hochberg correction was used to control the FDR. FDR ≤ 0.05 was used as the significance threshold. A linear mixed-effect model with a Tukey–Kramer HSD post hoc analysis was used to determine statistical significance of alterations in haematological parameters using JMP 13 Pro. A paired t test was used to evaluate if the changes in EPO levels and GATA1/GATA2 target gene expression were changed across infection stages. For the GATA1/GATA2 target gene analysis, SNM-transformed data was used. Each gene was averaged across the four animals and the significance test performed on the averaged values.

### Data release

Clinical data used in this analysis are publicly available in Plasmo DB and described in Joyner et al. [9]. The BM transcriptome data has been publicly deposited in GEO (Accession Number GSE94273).

## Results

### Insufficient compensation for anaemia during acute cynomolgi malaria in rhesus macaques

The parasitological and haematological data used in this analysis were published previously and made publicly available in Joyner et al. [9]. Briefly, five rhesus macaques were infected with *P. cynomolgi* M/B strain sporozoites and followed for up to 100 days. One macaque developed severe disease and irreversible kidney failure that required euthanasia; thus, this animal was removed from the current analysis [9]. Seven BM samples were collected longitudinally from each macaque, and these samples were classified into the following infection points for analysis in this manuscript: prior to inoculation (pre-infection), during acute primary infection, after the peak of parasitaemia (i.e. post-peak), before and between relapses (i.e. inter-relapses), and during relapses (Fig. 1a).

The rhesus macaques infected with *P. cynomolgi* M/B strain developed varying levels of anaemia during the primary infection (Fig. 1b; [9]). Despite the significant decrease in haemoglobin levels during acute infections, the circulating reticulocytes did not increase significantly until after the peak of parasitaemia or after sub-curative blood-stage treatment had been administered (Fig. 1b). These results suggested that the BM was not appropriately responding to the anaemia during acute infections but was restored after the peak of parasitaemia. Interestingly, relapses did not result in changes in haemoglobin levels or peripheral reticulocyte counts (Fig. 1b).

To assess whether each animal was appropriately compensating for anaemia, the reticulocyte production index (RPI) was calculated as previously described [9]. The RPI adjusts for alterations in reticulocyte maturation time in an anemic individual and provides a metric to assess if an appropriate compensatory response is being made by the BM to combat anaemia. An RPI value of under 2 when haemoglobin levels are decreasing suggests that the BM is not mounting an appropriate compensatory response for anaemia whereas an RPI of greater than 3 when haemoglobin is decreasing indicates an appropriate compensation. In support of the haemoglobin and reticulocyte data, there was not an appropriate compensatory response by the BM during acute infections (Fig. 1b). However, an appropriate response was mounted after the peak of parasitaemia and treatment, though haemoglobin levels continued to decrease (Fig. 1b).

Erythropoietin (EPO) is critical for inducing erythropoiesis during normal RBC turnover and pathological conditions. Thus, the lack of response by the BM during acute infection could have been due to dysregulation of erythropoietin production. In contrast to this hypothesis, EPO levels were significantly increased during acute infection (Fig. 1c). Collectively, these analyses demonstrated that there was not an appropriate compensatory response by the BM in the face of the developing anaemia early during acute infections.

### Acute malaria, but not relapses, leads to substantial changes in the bone marrow transcriptome

The next goals of this study were to analyse the transcriptional changes in the BM that may account for the lack of compensatory response by the BM during acute infection and to determine if relapses induced changes in the BM since anaemia was not observed during relapses. RNA-Seq was performed using BM MNCs from the four rhesus macaques infected with *P. cynomolgi* at the time points indicated in Fig. 1a and classified for downstream analysis as described above.

Hierarchical clustering was performed with the R mixOmics package [9] to determine the similarity and/or differences in the BM transcriptome among the animals for each infection point. Using this approach, the acute infection measurements clustered together, but no other infection points clustered with a discernable pattern, suggesting that factors other than the BM transcriptome were influencing the grouping of these stages (Additional file 1A). After determining that 26.7% of the gene expression variance was attributable to each individual, the variance associated with each individual was removed using a supervised normalization of microarray (SNM) transformation (Additional file 1B).

**Fig. 2:**
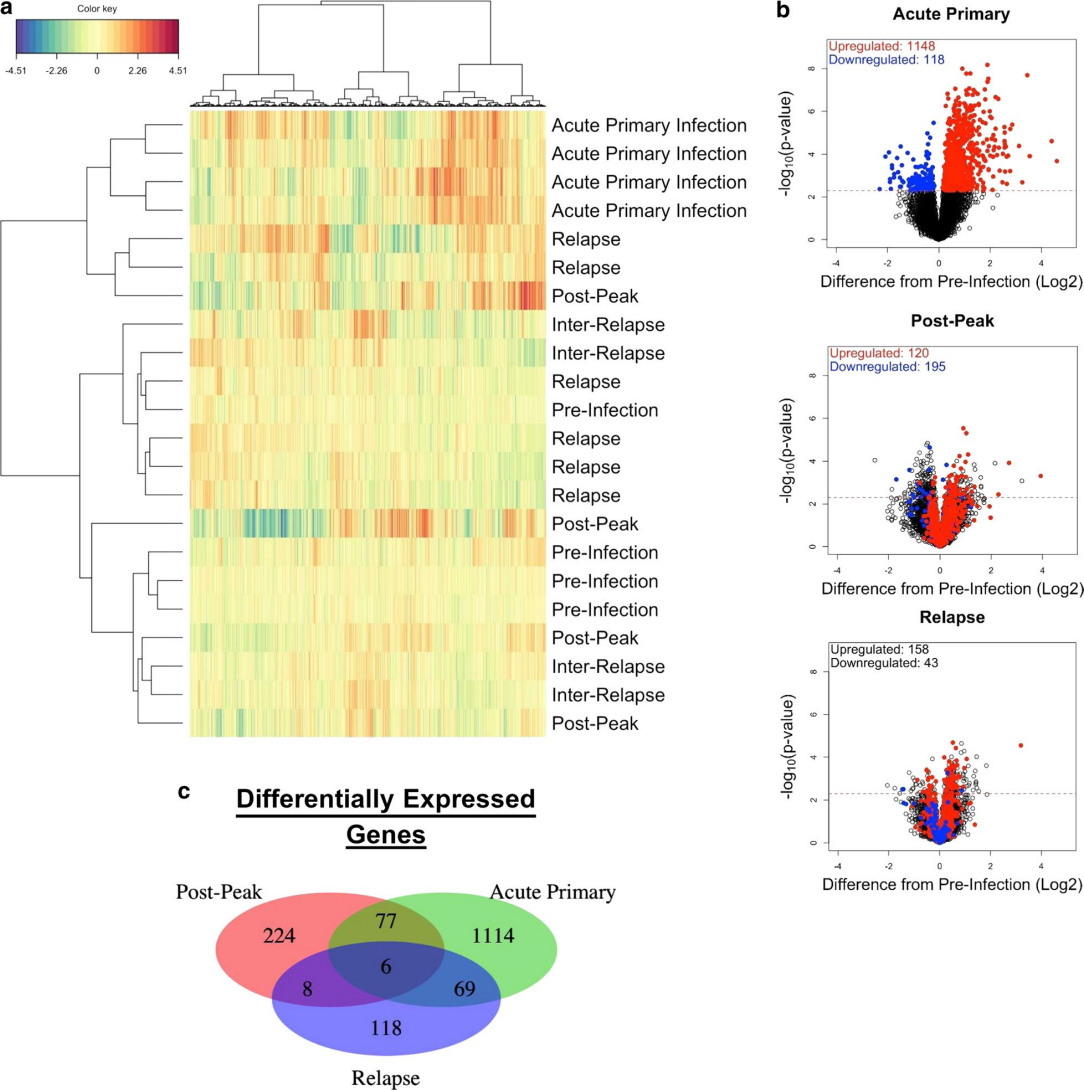
Acute malaria but not malaria relapse causes substantial changes in the bone marrow transcriptome. **a** Clustered heatmap of the BM transcriptome, with infection points of samples indicated. Four samples from acute primary infection form a cluster separated from other infection points. Another cluster captures more mild infection responses, including four out of six relapse time points. Colours indicate z-score normalized expression values. **b** Volcano plots of differentially expressed host genes in the BM at acute primary infection, post-peak, and relapse compared to pre-infection. The y axis is the negative logarithm base 10 of the p value. The x axis is the log2 difference in expression values between pre-infection and the infection point of a given plot. The red dotted horizontal line represents FDR = 0.05. Thus, the right arm of dots above the threshold line corresponds to genes significantly upregulated compared to pre-infection; the left arm of dots above the threshold line corresponds to downregulated genes. Red and blue dots for all three plots represent genes upregulated or downregulated (respectively) at acute primary infection, visualizing the fact that few genes differentially expressed at primary infection are differentially expressed during post-peak and relapse, with many genes not even trending in the same direction between the two infection points. **c** Venn diagram showing the overlap of differentially expressed genes at acute primary infection, post-peak and relapses

After transformation, 42.3% of the variance of the dataset was now attributable to the infection point (Additional file 1C). Hierarchical clustering of the data after accounting for the variance attributable to individual animals maintained clustering of the acute infection stage (Fig. 2a). However, this also led to a more robust clustering of the other infection stages, although the clusters were not completely correlated with infection points (Fig. 2a). This suggested that acute malaria caused significant and coherent changes in the BM transcriptome, but there did not appear to be unique changes during relapses, post-peak, or inter-relapse infection periods.

Since hierarchical clustering indicated that transcriptional profiles at post-peak and inter-relapse infection points were generally similar to pre-infection profiles, similarities and differences were formally evaluated between each infection point. The number of differentially expressed genes (DEGs) between infection points was determined using analysis of variance (ANOVA). Compared to pre-infection, 1266 genes were differentially expressed during acute infection, 315 after the peak of parasitaemia, and only 201 genes during relapses (Fig. 2b). For relapses, 75 of these genes were in common with acute infection (Fig. 2c). During acute infection, 1148 of the DEGs were upregulated whereas 118 were downregulated compared to pre-infection. Relapses showed much less variance in gene expression even for genes with large absolute upregulation and high significance during acute infection. Thus, while acute infection caused significant perturbations in the BM transcriptome, relapses had a small effect.

### Pathways and processes altered in the bone marrow during acute malaria

Gene ontology analysis was employed to understand the pathways and processes altered during acute infection. Approximately 37 biological processes were significantly changed in the BM during acute infection (Additional file 2). The identified pathways could largely be consolidated into biological processes related to cellular stress (e.g. response to endoplasmic reticulum stress), increases in transcription (e.g. RNA processing), and ongoing immune responses in the BM (e.g. Type I IFN, IFNγ, cell response to cytokine signaling, etc.). The pathways and processes of the other infection points were also analysed but none were significantly enriched; this is likely due to the low number of differentially expressed genes during these infection stages.

**Fig. 3:**
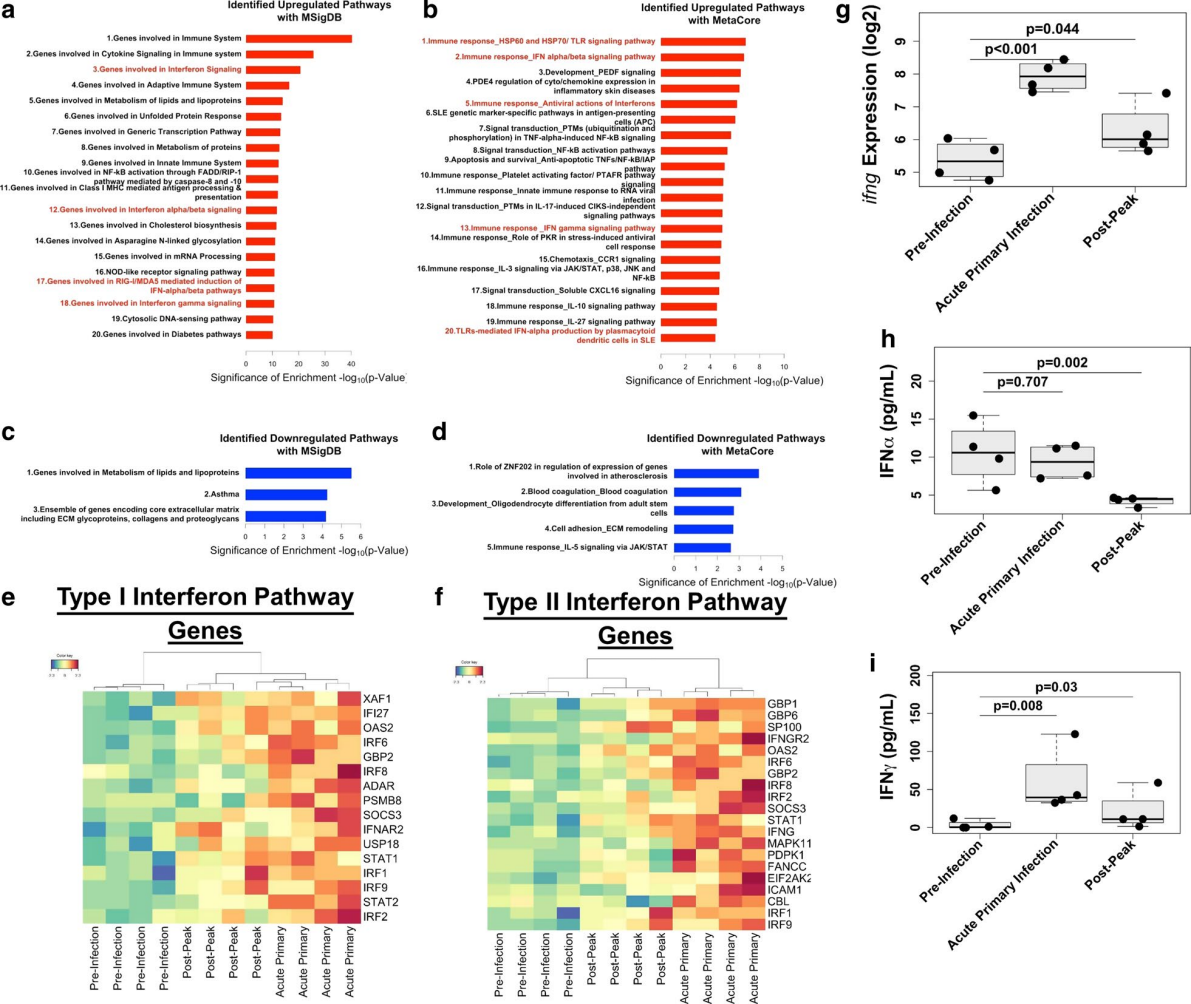
Type I and Type II interferon transcriptional signatures are enriched in the bone marrow during acute malaria. **a** Pathways enriched with upregulated genes using GSEA. Pathways associated with interferons are highlighted in red. Bars indicate the negative logarithm base 10 of the p value of the enrichment for a given gene set. **b** Pathways enriched with upregulated genes using MetaCore. Pathways associated with interferons are highlighted in red. **c** Pathways enriched with downregulated genes using GSEA. **d** Pathways enriched with downregulated genes using MetaCore. **e** Clustered heatmap of transcriptional profiles of Type I IFN signature genes. Colours indicate z-score normalized expression values. **f** Clustered heatmap of transcriptional profiles of Type II IFN signature genes. **g** *ifnγ* BM expression levels. **h** IFNα serum concentrations. **i** IFNγ serum concentrations

Pathway analysis using MetaCore (Thomson Reuters) and Molecular Signatures Database (MSigDB) [29, 30] identified additional molecular pathways that were increased during acute infection (Fig. 3). The pathways identified were similar between both databases and were skewed towards upregulation (Fig. 3a–d). This is likely due to the low number of down-regulated DEGs during acute infection (Fig. 2b). Full lists of pathways identified in each database are provided in Additional files 3, 4, 5 and 6.

The enriched pathways and gene sets from both databases highlighted the ongoing immune response in the BM during acute infection; in particular, pathways involved in cytokine signaling were overrepresented (Fig. 3a, b). The most predominant cytokine signatures to emerge from the analysis of acute infection in both databases were Type I (i.e. IFN-α/β) and Type II interferon (i.e. IFN-γ), which were identified by both databases (Fig. 3a, b). A heatmap of DEGs identified in these pathways shows upregulation during acute infection but not other infection points (Fig. 3e, f). Although the related pathway was identified, changes in expression of the *ifnα* and *ifnβ1* genes themselves were not captured by RNA-Seq during acute infection because the genes did not pass the FPKM threshold. *ifnγ* expression, on the other hand, was detectable and significantly increased (p < 0.001) during acute infection and after the peak of parasitaemia (p < 0.05) (Fig. 3g). To ensure that gene expression was indicative of the protein levels, the concentrations of IFNγ and IFNα in the plasma were measured via multiplex assay. Consistent with the transcriptional data, IFNγ levels were increased (p = 0.008; Fig. 3i) and IFNα levels did not change during acute infection although a significant decrease was detected after the peak of parasitaemia (Fig. 3h).

Other cytokine signatures identified as transcriptionally upregulated in the BM during acute infection included IL-10 and IL-27, which are known to have effects on the BM during acute malaria, and genes involved in the IL-3 signalling pathway (Fig. 3b) [31, 32]. Interestingly, the IL-5 signalling pathway was the only cytokine signalling pathway identified as being downregulated during acute infection, which could be due to the skewing towards a Th1 response (Fig. 3d).

This analysis also revealed the enrichment of pathways associated with pathogen recognition receptors (PRRs) that are important for sensing intracellular and extracellular microbial components as well as host danger molecules. Specifically, the analysis highlighted the importance of engagement of Toll-like Receptors, NOD-Like receptors and RIG-I/MDA5 (Fig. 3a, b).

### Intermediate and non-classical monocytes may negatively impact the erythroid lineage during acute malaria

Bone marrow is made up of a heterogeneous population of cells that together yield the transcriptional profiles measured here. Weighted Gene Set Co-expression Network Analysis (WGCNA) was employed to investigate which cells types in the BM may potentially be responsible for the observed changes in the BM transcriptome during acute malaria [33]. WGCNA was performed using DEGs identified during the acute infection and flow cytometry data collected at each infection point. The gating strategy for the cellular subsets used in WGCNA analysis is shown in Additional file 7. The populations included in the analysis were as follows: classical monocytes, intermediate monocytes, non-classical monocytes, B-cells, T-cells, granulocytes and a mixed population of erythroid progenitors (Additional file 8). Samples from other infection points were not included in this analysis to allow the acute infection to drive the formation of the coexpression modules.

**Fig. 4:**
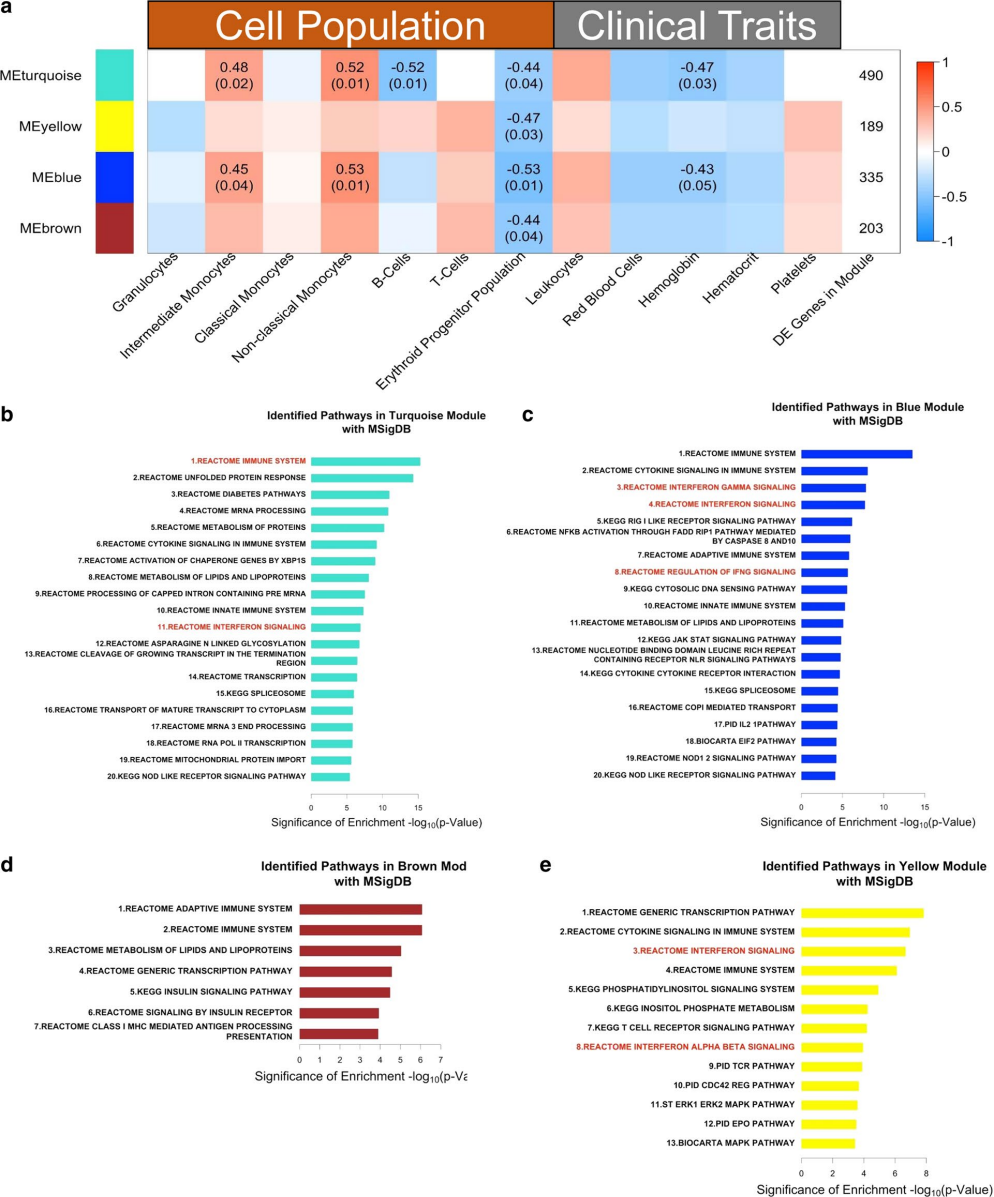
Intermediate and non-classical monocytes in the bone marrow are correlated with gene modules that may negatively impact the erythroid lineage. **a** Correlation of gene modules with cell population measurements and clinical traits based on WGNCA analysis of the BM transcriptome. Rows contain different transcriptional modules identified by WGCNA, with the colour in a given column indicating the degree of correlation of that module with cell population or clinical trait measurements. The top number in each entry is the Spearman correlation coefficient for any correlation with p < 0.05, and the bottom number is the p value significance of the correlation coefficient. The last column indicates the number of genes in each module. **b**–**e** Significantly enriched pathways in the turquoise, blue, brown, and yellow modules based on analysis using MSigDB. Bars indicate the negative logarithm base 10 of the p value of the enrichment for a given gene set

The dominant modes of variance of each module were used to evaluate the association of the modules with cellular subsets. WGCNA identified four gene coexpression modules (Fig. 4a). The modules coloured turquoise and blue were composed of 490 and 335 DEGs, respectively, and were positively correlated with the number of intermediate and non-classical monocytes (p < 0.05) in the BM. The turquoise module was negatively correlated with B cell numbers (r = −0.52, p = 0.01). However, unlike monocytes, this was a negative correlation and is likely related to the disruption of the BM environment where B-cells develop. Other immune cell types identified by flow cytometry were not correlated with any of these co-expression modules. These results suggest that changes in the BM transcriptome during acute malaria may have been primarily related to monocytes.

To determine whether there was a link between the identified co-expression modules and suppression of the erythroid progenitor population, the relationship of the co-expression modules with the erythroid progenitor population was examined. All four co-expression modules were correlated with a drop in erythroid progenitors (p < 0.05 in all cases). Since only the turquoise and blue modules were associated with immune cells (monocytes), this suggested that the correlation of the yellow and brown co-expression modules represented non-immunological or diffuse immunological pathways.

### Intermediate and non-classical monocytes are associated with pathways upregulated during acute malaria in the bone marrow

To test the hypothesis that monocytes were a major driving force of the transcriptome changes in the BM, pathway enrichment analysis was first performed on the genes in each module [29, 30]. The MSigDB database was used for this analysis; results obtained with this database were comparable to those obtained using MetaCore (Fig. 4b–e). The turquoise module was enriched with pathways involved in the immune response (and interferon signalling pathways), general cellular response pathways (e.g. unfolded protein response), and RNA metabolism (Fig. 4b). The blue module was enriched in pathways related to the immune response, including cytokine signalling by IFNγ, activation of NF-κB, and the IL-2 pathway (Fig. 4c). Each of these pathways are known to be involved in monocyte activation [34]. This is consistent with the hypothesis that the activation status of the intermediate and non-classical monocyte populations in the BM during acute infection may have influenced the BM transcriptome. Indeed, the intermediate and non-classical monocyte populations were expanded in the marrow during acute infection, but these differences did not reach statistical significance likely due to the sample size (Additional file 9).

The blue module was also enriched for the PRR pathways activated through NOD like receptors (NLRs) and RIG-I like receptors (RLRs) (Fig. 4c). This suggests that these pathways, which were originally identified by DEG analysis, may be active in intermediate and non-classical monocytes in the BM in response to *Plasmodium* infection.

**Fig. 5:**
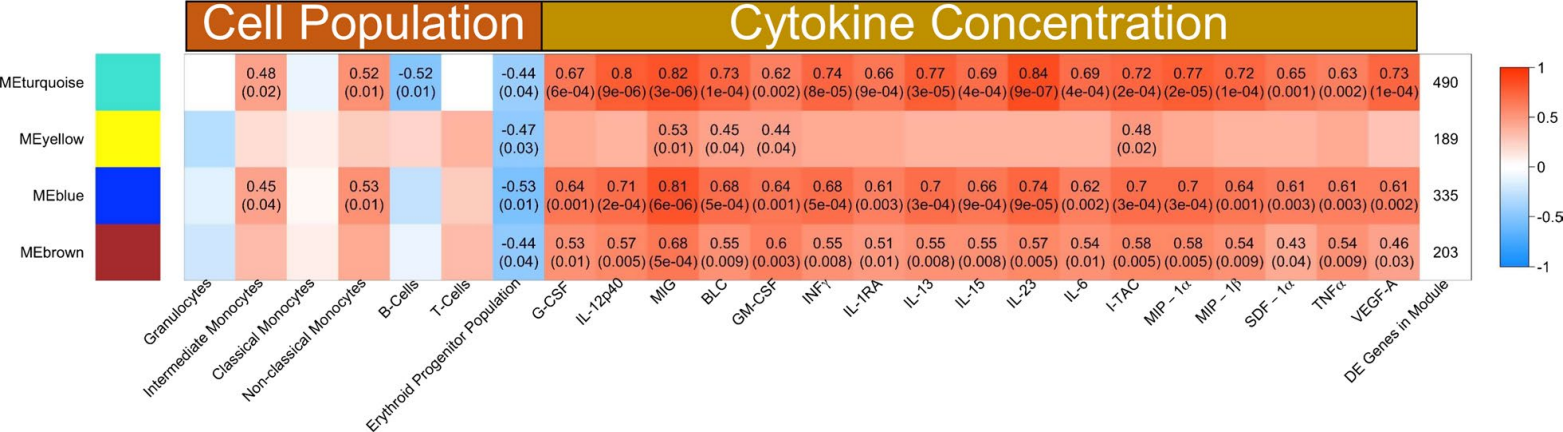
Systemic cytokines are positively associated with transcriptional modules that are negatively associated with erythroid progenitors but positively correlated with intermediate and non-classical monocytes. The correlation of gene modules with cell population measurements and cytokine concentrations based on WGNCA analysis of the BM transcriptome is shown. Cytokine measurements were performed using a multiplex assay with plasma collected after isolation of bone marrow mononuclear cells collected for RNA-Seq analysis. Rows contain different transcriptional modules identified by WGCNA, with the colour in a given column indicating the degree of correlation of that module with cell population or cytokine measurements. The top number in each entry is the Spearman correlation coefficient for any correlation with p < 0.05, and the bottom number is the p value significance of the correlation coefficient. The last column indicates the number of genes in each module

To validate the transcriptomic signatures related to cytokine signalling and identify the cytokines that may negatively impact the erythroid progenitors, WGCNA analysis was repeated using the flow cytometry, transcriptomics and cytokine data. Seventeen of 45 cytokine concentrations were found to be positively correlated with the turquoise and blue modules. Transitively, these cytokines were thus also negatively associated with the erythroid progenitor population (Fig. 5). Although negatively correlated with the erythroid population in the BM, these cytokines were positively associated with the frequency of non-classical and intermediate monocytes (Fig. 5). IFNγ was correlated with the blue, brown, and turquoise modules, and IFN signalling was enriched in the turquoise and blue modules, which provided confirmatory support for the transcriptome analysis and the validity of the WGCNA approach (Fig. 4a, c).

The brown module did not correlate with particular immune cell types, and this is likely due to the diverse set of pathways represented in this module (Fig. 4d). The yellow module highlighted IFN signalling pathways (Fig. 4e) but was not correlated with protein levels of IFN. Interestingly, a signature driven by erythropoietin (EPO) was also enriched in the yellow module (Additional file 10). Although an EPO signature is to be expected given that EPO plays an important role to increase BM output of RBCs during anaemia, this signalling was inversely correlated in this study with the erythroid progenitors, suggesting that despite the elevated EPO levels in the plasma and signalling in the BM, an appropriate compensatory response could not be made. Indeed, these data are consistent with previous literature showing that malaria induces an EPO-independent anaemia [35].

### Transcriptional networks related to erythropoiesis are disrupted in the bone marrow during acute malaria

**Fig. 6:**
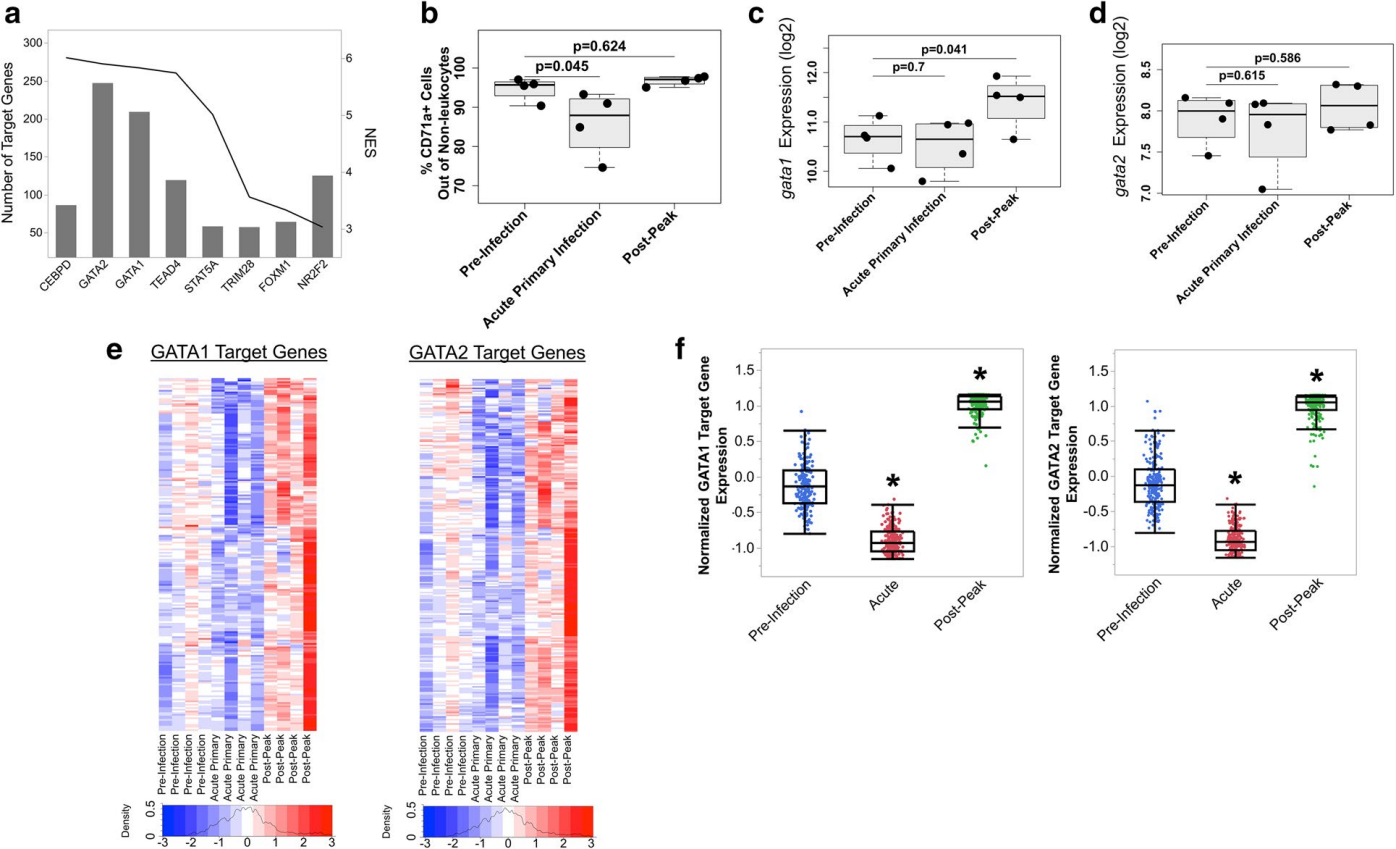
Disruption of GATA1 and GATA2 in erythroid progenitor cells may contribute to the decrease in erythroid progenitors and insufficient erythropoietic output during acute malaria. **a** Transcription factor analysis identified eight transcription factors (NES > 3) associated with the cluster of genes positively correlated with reticulocyte levels. The line plot indicates the NES score, while the bar plot indicates the number of genes in the set containing a binding site for each transcription factor. **b** Frequency of erythroid progenitors in the bone marrow during different infection periods as determined by flow cytometry. There is a small but statistically significant decrease at acute primary infection. **c**, **d** Transcriptional expression levels of GATA1 and GATA2 in BM. Expression of GATA1 was upregulated at post-peak, but not at acute primary infection, while GATA2 levels were not significantly changed. **e** Heatmap of gene expression levels of the GATA1 and GATA2 transcriptional regulatory network; samples are in chronological order, while genes are hierarchically clustered. Most pathway members were downregulated at acute primary infection and upregulated at the post-peak infection point. **f** Gene expression of GATA1/2 targets was downregulated at acute primary and upregulated at post-peak compared to pre-infection. Gene measurements were averaged across animals for each infection point, and paired t tests were used to assess statistical significance (Asterisk: significant at p < 0.01)

Given insufficient compensation for anaemia during acute malaria, the transcriptome data was mined to determine if transcriptional networks related to erythropoiesis were disrupted during acute *P. cynomolgi* infections. Correlation analysis (CA) was performed using the erythroid population frequencies determined by flow cytometry and BM MNC gene expression to identify genes that may be related to the decrease in erythroid progenitors observed in the BM (Fig. 6b). Samples from pre-infection, acute primary, and post-peak were utilized for this analysis to determine changes that occurred before, during, and after the onset of anaemia.

547 genes from the same RNA-Seq dataset utilized in the previous analyses were identified as positively correlated (Spearman’s correlation coefficient >0.6; p < 0.05) at FDR < 0.2 with the frequency of the erythroid progenitor population in the BM (Additional file 11). After identifying the related genes, the iRegulon pipeline was utilized to explore what transcription factors may be influencing the genes that were positively correlated with the change in erythroid progenitor cell frequencies during acute infection. iRegulon identified multiple transcription factors including CCAAT/Enhancer Binding Protein (CEBPD), GATA Binding Proteins (GATA1/GATA2) and TEA Domain Transcription Factor 4 (TEAD4) as having their targets enriched in the genes that correlated with erythroid progenitor cell frequencies (Fig. 6a).

This analysis focused on GATA1 and GATA2 because these proteins are master regulators of erythropoiesis, had the largest number of target genes identified in the dataset, and had two of the highest NES scores, which are a metric of statistical significance in iRegulon (Fig. 6a) [36, 37]. First, the expression of GATA1 and GATA2 was examined directly to understand if the gene expression of these proteins was changed during infection, which could directly impact the erythropoietic response during infection. GATA2 gene expression did not change throughout the initial infection (Fig. 6d). In contrast, GATA1 gene expression was not changed during acute infection but was significantly increased after the peak of parasitaemia when erythropoiesis was restored (Fig. 6c). Thus, transcription of GATA1/GATA2 did not appear to completely or directly explain the correlation between erythroid progenitors and hundreds of genes that are their targets, though the increases in GATA1 and progenitor levels at post-peak suggest GATA1 transcription could still be involved.

Next, the genes regulated by GATA1 and GATA2 were examined to identify whether the genes regulated by these proteins were differentially regulated even though transcription of GATA1/GATA2 was not altered significantly during acute infection. The target genes of GATA1 and GATA2 correlated with the small but significant decrease (p = 0.045) in the frequency of the erythroid progenitor population in the BM during acute infection (Fig. 6b). Notably, the expansion of other cell types in the marrow during acute infection could drive this decrease in percentage of erythroid progenitors. Importantly, the genes regulated by GATA1 and GATA2 were downregulated during acute infection, but upregulated post-peak when the erythroid progenitor population in the BM was restored to pre-infection levels and an increase in peripheral reticulocytes was observed, indicating restored erythropoiesis (Fig. 6e, f). Therefore, direct suppression of GATA1/GATA2 transcription does not appear to be responsible for the lack of compensatory erythropoiesis in the periphery, and instead, it seems that these proteins’ functions may be compromised at the post-translational level during acute infection.

## Discussion

The development of malarial anaemia is multi-factorial, and it is clear that the loss and sustained reduction of RBCs during an infection is not due to parasitism alone. Bone marrow suppression and the removal of uninfected RBCs contribute to the development of anaemia. In the rhesus macaque—*P. cynomolgi* model, both processes are active, based on haemoglobin and reticulocyte kinetics described here and in Joyner et al. [9]. Interestingly, insufficient erythropoiesis appears to contribute to the development of anaemia early during a blood-stage infection since appropriate compensation for anaemia is observed after the peak of parasitaemia or after the administration of blood-stage treatment (Fig. 1). Furthermore, there were few changes in the BM transcriptome after the peak of parasitaemia, suggesting BM is no longer dysregulated (Fig. 2). Thus, it is clear that there are multiple phases in the development of malarial anaemia in macaques infected with *P. cynomolgi*. In each phase, there are different pathological processes that may predominate, and future studies should aim to explore the molecular mechanisms underlying each phase, particularly the removal of uninfected red blood cells.

Predictions from field studies have suggested that relapses are responsible for most *P. vivax* blood-stage infections and potentially most clinical illness [38–40]. Therefore, the initial hypothesis of this study was that both *P. cynomolgi* acute primary infections and relapses would cause alterations in the BM transcriptomes since anaemia would be an expected complication in both cases. However, unlike the acute infections, relapses did not result in significant changes in the BM transcriptomes. Although unexpected, this finding is in agreement with the conclusions in Joyner et al., which demonstrated that *P. cynomolgi* relapses are not associated with the development of anaemia, even when peripheral parasitaemia is detectable [9].

The lack of anaemia and changes in the BM transcriptome during *P. cynomolgi* relapses is likely due to the significant decrease in parasite burden during relapses in comparison to the initial infection (Fig. 1). This decrease is likely due to the development of immunity that prevents the parasite from reaching levels similar to those during an initial exposure. Lower levels of parasite replication may allow the BM to maintain its normal compensatory functions during relapses, unlike acute infection where insufficient erythropoietic output is linked to higher parasite densities [9]. Indeed, there is evidence from malaria chemotherapy studies that relapses caused by *P. vivax* also do not necessarily result in disease, and thus, this phenomenon does not seem to simply be attributable to nonhuman primates [41–43]. Taken together, this evidence suggests that there is a threshold of parasite burden that disrupts bone marrow function and erythropoietic output prior to the development of effective anti-parasite immunity.

During acute infection, many biological pathways and processes in the BM were upregulated, including pathways related to cellular metabolism and transcription. Such pathways are likely representative of the ongoing host response against the parasite both in the periphery and potentially in the BM since both *P. vivax* and *P. falciparum* iRBCs can readily be found in the BM [44, 45]. Many of the upregulated pathways in the BM were related to inflammation and largely composed of cytokine signalling pathways. This agrees with previously published work demonstrating that cytokines, including IL-10 and IL-27, are important in BM responses during rodent malaria and/or in malaria patients [32, 46]. The current data suggest that these cytokines may also be involved in the BM of NHPs with malaria.

Here, Type I and Type II IFN signalling pathways were identified as potentially key cytokines involved in malarial anaemia. However, despite an enrichment in transcriptional pathways involved in Type I IFN signalling in the BM, IFNα protein levels in the plasma were not increased (Fig. 3h). (*ifnα* gene expression in the BM was not reliably quantified.) In stark contrast, the Type II IFN signature was accompanied by an increase in *ifn*γ gene expression in the BM and IFNγ concentration in the plasma, and the erythroid progenitor population was negatively correlated with IFNγ (Figs. 2i, 3g, 4). These data suggest that Type II IFNs could negatively impact the erythroid progenitor population through direct or indirect mechanisms during acute infection. Indeed, IFNγ can directly cause apoptosis of erythroid progenitors in vitro and it has been previously implicated in malarial anaemia in rodent malaria models [12, 47, 48].

Type I IFNs can work in concert with IFNγ to suppress erythropoiesis [49–51], and therefore it is possible that Type I IFNs may initiate the disruption of erythropoiesis earlier during infection. The reasons why Type I IFN transcripts were too low to be quantified could be because they are more tightly regulated than IFNγ and the sampling regimen missed the point at which these proteins are synthesized and released into the plasma. Alternatively, these proteins may act predominantly at local levels and may have only been present in the BM. There also may be other intermediary molecules that are unmeasured here but may have a role in the observed phenomena. Regardless, recent studies demonstrate Type I IFNs to be important during early blood-stage malaria, and the results presented here suggest these cytokines should be explored further in relation to malarial anaemia [52–54].

This study’s results also suggested the possibility that monocytes were largely responsible for changes in the BM transcriptome during acute infection (Figs. 3, 4). It has previously been shown that macrophages and monocytes in the BM produce inflammatory cytokines in response to parasite byproducts such as haemozoin. It has been hypothesized that this process drives the dyserythropoiesis observed during malarial anaemia [55]. Although most of these conclusions were drawn from in vitro experiments, the analysis here supports a model where monocytes in the BM negatively influence the erythroid lineage in vivo via cytokine production during acute malaria [55]. It is interesting to speculate that these signatures may be coming from BM-resident monocytes and/or macrophages, but more work will be required to explore this possibility in future studies.

Although intermediate and non-classical monocytes are thought to be important for parasite control in the periphery [56], this analysis from the BM implicated these monocytes as potentially being responsible for the decrease in the erythroid progenitors. WGCNA analysis associated these monocyte subsets with an increase of pro-inflammatory cytokines such as IFNγ, MIP1-α/β and TNFα in the plasma, which would not necessarily be expected of these monocytes, as these subsets are not considered to be classically pro-inflammatory. Indeed, many of these cytokines are known to negatively impact erythroid progenitors, directly or indirectly [12, 55]. It is interesting to speculate that these monocytes may play a dual role in controlling parasite growth through cytokine production, etc. in the peripheral blood but also contribute to the development of anaemia through these same processes. Future work could assess this directly using this NHP model of *P. vivax* infection.

Previous evidence has suggested that the disruption of erythropoiesis may be due to dysregulation of transcription factors that control erythropoiesis [57]. In this study, it was shown that GATA1 and GATA2, two master regulators of erythropoiesis, may not function appropriately during acute malaria. Genes regulated by GATA1 and GATA2 were downregulated during acute infection, but upregulated whenever appropriate erythropoietic output was restored. Indeed, some of the cytokines (e.g. TNFα, IFNγ) that were upregulated are known to antagonize GATA1 and, thus, disrupt terminal erythroid differentiation [58, 59], providing a potential mechanism for what was observed. Although both GATA1 and GATA2 were identified, GATA1 may be more central in the process, based on *gata2* gene expression not being upregulated when erythropoietic output was restored (Fig. 5g). Future studies should examine the factors that influence the function of GATA1/GATA2 during malaria since it is clear that a variety of intermediate molecules besides those described here could also affect each protein’s function [12, 60, 61].

Although this study provides the basis for future investigations related to malarial anaemia using NHPs, there are limitations. First, the number of NHPs presented in this study is smaller than initially designed because one macaque from the cohort developed severe disease during the acute infection period and required euthanasia [9]. This decrease in the cohort size may limit the generalizability of the results, but other studies using in vitro systems, rodent malaria models, and samples from malaria patients have arrived at conclusions similar to those in this study [12, 55]. This suggests that studies with NHPs, which in comparison to studies using patient samples or rodent models will be restricted to small sample size, have the potential to produce generalizable results.

A second limitation of this study is that the transcriptomic profiling of the BM by RNA-Seq was performed on BM MNCs. This sample type is composed of multiple cellular lineages, and thus it is difficult to pinpoint the specific cell type in the BM that was responsible for changes in the transcriptome. The changes identified here are thus necessarily correlative rather than describing definitive and direct mechanisms in specific cell types. Although such uncertainty is in some ways a weakness, this approach also has its advantages. Most importantly, it enabled an unbiased survey of the major changes in the BM during acute malaria and relapse infections in macaques. In contrast, an initial focus on a singular cell type would likely have missed some critical transcriptional changes in the BM, and thus the insights derived from those changes. Future studies can target specific cellular lineages to better understand the role of each individual cell type in the BM during infection.

## Conclusion

This NHP cohort study has direct implications for understanding *P. vivax* malaria and possibly other *Plasmodium* species. Insights have been gained into BM dysfunction and inflammatory processes during the acute stage of the disease caused by *P. cynomolgi* in rhesus macaques. Specifically, monocytes were identified as a potentially critical cell type involved in inflammation in the BM during acute malaria in macaques. Furthermore, monocytes were associated with a decrease in erythroid progenitors in the BM, suggesting monocyte-driven inflammation could suppress erythropoiesis in vivo. While erythroid progenitors were decreasing, GATA1 and GATA2 transcriptional networks, which are critical for terminal erythroid differentiation, were perturbed. This study provides a potential explanation for insufficient erythropoiesis during acute malaria in primates and paves the way for future investigations that can assess the role of specific cell types and pathways in this context. Such studies will be critical for new experimental directions aimed at identifying targets of possible interventions for malarial anaemia. This study also shows for the first time that malaria relapses, shown earlier not to be associated with clinical illness in this model, do not cause similar perturbations of the BM as observed during primary acute infections.

## Additional files

**Additional file 1.** Overview of the BM transcriptome prior to SNM transformation. (A) Clustered heatmap of the BM transcriptome before animal effects were removed. Four samples of acute primary infection form a cluster separated from other infection points. Other infection points do not form unique or separated clusters, with many of the most closely related samples coming from the same animals, suggesting that individual effects may confound the ability to identify the effect of infection points on gene expression profiles. Colours indicate z-score normalized expression values. (B) Variance Component Analysis showing that individual animal effects originally explain 26.7% of the gene expression variance, while infection points explain 22.4% of the gene expression variance. (C) After removing individual effects by SNM, infection points explain 42.3% of the gene expression variance.

**Additional file 2.** Gene ontology processes that are altered in the bone marrow during acute *P. cynomolgi* infections of rhesus macaques. (FDR B&H: FDR corrected with Benjamini–Hochberg procedure; FDR B&Y: FDR corrected with Benjamini–Hochberg–Yekutieli procedure).

**Additional file 3.** Complete list of gene sets that are upregulated in the bone marrow during acute *P. cynomolgi* infections of rhesus macaques using the GSEA database.

**Additional file 4.** Complete list of pathways that are upregulated in the bone marrow during acute *P. cynomolgi* infections of rhesus macaques using the Metacore database.

**Additional file 5.** Complete list of gene sets that are downregulated in the bone marrow during acute *P. cynomolgi* infections of rhesus macaques using the GSEA database.

**Additional file 6.** Complete list of pathways that are downregulated in the bone marrow during acute *P. cynomolgi* infections of rhesus macaques using the Metacore database.

**Additional file 7.** Flow cytometry gating strategy to monitor cellular subset in the bone marrow of rhesus macaques with malaria. A representative gating strategy for determining the frequency of various immune cell subsets in bone marrow aspirate collected from rhesus macaques during *P. cynomolgi* infection. This representative plot is from a pre-infection time-point.

**Additional file 8.** Characterization of the cellular subsets constituting the erythroid progenitor cell population. (A) FACS profiles corresponding to (B). (B) Cells consistent with the cell surface phenotype of erythroid progenitors were identified based on CD34 and CD71a surface staining.

**Additional file 9.** Changes in monocyte subsets in the bone marrow during an initial blood-stage *P. cynomolgi* infection. The percentage of classical (CD14+CD16−), intermediate (CD14+CD16+), and non-classical (CD14−CD16+) monocytes out of the monocyte compartment are shown before infection, during the acute primary infection, and after the peak of infection.

**Additional file 10.** Transcriptional profiles of genes in the EPO pathway before, during, and immediately after acute malaria. The genes in the erythropoietin pathway identified as differentially expressed in the bone marrow RNA-Seq data set are shown. Samples are hierarchically clustered. Colours indicate z-score normalized expression values.

**Additional file 11.** List of genes identified as significantly positively correlated (Spearman’s correlation coefficient >0.6; p < 0.05) at FDR < 0.2 with the frequency of the erythroid progenitor population in the BM.

**Additional file 12.** Erythropoietin ELISA results from *P. cynomolgi* B strain infections of rhesus macaques. RFv13, the individual that succumbed to the infection, is included in this file although the data is not presented in the manuscript.

## Abbreviations

ANOVA: analysis of variance
BM: bone marrow
CA: correlation analysis
CEBPD: CCAAT/Enhancer Binding Protein
DEA: differential expression analysis
DEGs: differentially expressed genes
EPO: erythropoietin
FACS: fluorescence-activated cell sorting
FBS: Fetal Bovine Serum
FDR: false discovery rate
FPKM: fragments per kilobase of transcript per million
GSEA: gene set enrichment analysis
iRBCs: infected red blood cells
MNCs: mononuclear cells
NHP: non-human primate
NLRs: NOD like receptors
PRR: pattern recognition receptor
rbc: red blood cell
RLRs: RIG-I like receptors
SNM: supervised normalization of microarrays
TEAD4: TEA Domain Transcription Factor 4
WGCNA: weighted gene co-expression network analysis
YNPRC: Yerkes National Primate Research Center
