# Correction to: Integrative analysis associates monocytes with insufficient erythropoiesis during acute *Plasmodium cynomolgi* malaria in rhesus macaques

**DOI:** 10.1186/s12936-017-2134-z

**Published:** 2017-12-04

**Authors:** Yan Tang, Chester J. Joyner, Monica Cabrera-Mora, Celia L. Saney, Stacey A. Lapp, Mustafa V. Nural, Suman B. Pakala, Jeremy D. DeBarry, Stephanie Soderberg, the MaHPIC Consortium, Jessica C. Kissinger, Tracey J. Lamb, Mary R. Galinski, Mark P. Styczynski

**Affiliations:** 1 School of Chemical & Biomolecular Engineering, Georgia Institute of Technology, Atlanta, GA USA; 2 Malaria Host–Pathogen Interaction Center, Emory Vaccine Center, Yerkes National Primate Research Center, Emory University, Atlanta, GA USA; 3 Institute of Bioinformatics, University of Georgia, Athens, GA USA; 4 Department of Genetics, University of Georgia, Athens, GA USA; 5 Center for Tropical and Emerging Global Diseases, University of Georgia, Athens, GA USA; 6 Department of Computer Science, University of Georgia, Athens, GA USA; 7 Department of Pathology, University of Utah, Salt Lake City, UT USA; 8 Division of Infectious Diseases, Department of Medicine, Emory University, Atlanta, GA USA

## Correction to: Malar J (2017) 16:384 10.1186/s12936-017-2029-z

After publication of the article [1], it was brought to our attention that several symbols were missing from Fig. 1, including some cited in the figure’s key. The correct version of the figure is shown below and has now been updated on the original article.

**Fig. 1:**
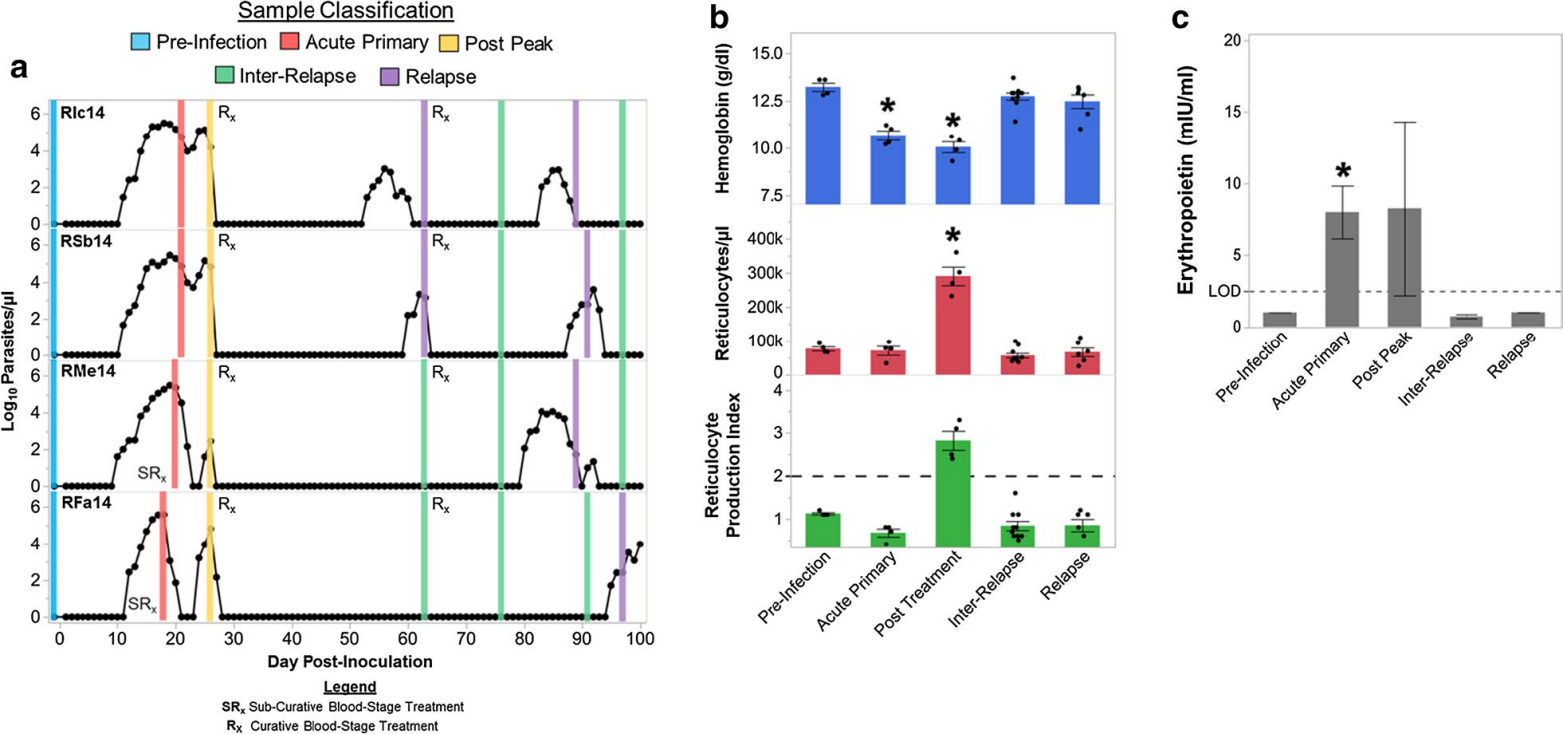
The bone marrow does not compensate for anemia during acute cynomolgi malaria despite increased EPO levels. **a** Parasitaemia kinetics for the four animals are shown. Bone marrow sample collection times in relation to parasite kinetics are indicated by a vertical bar; the colour of the bar indicates the infection point classification of the sample. **b** Hemoglobin levels, peripheral reticulocyte numbers, and the reticulocyte production index for each infection stage are shown. Statistical significance relative to pre-infection was assessed where relevant using a linear mixed-model with Tukey–Kramer post hoc analysis. **c** Mean erythropoietin levels during each infection stage are indicated. Dashed line indicates the limit of detection of the assay. Statistical significance was assessed using a paired t test relative to pre-infection levels. Error bars indicate standard error for **b** and **c**. Asterisk indicates p < 0.05
