# Supplementary figures and images for: Integrative analysis associates monocytes with insufficient erythropoiesis during acute Plasmodium cynomolgi malaria in rhesus macaques
Source: Malar J. 2017 Sep 22;16:384. doi: 10.1186/s12936-017-2029-z (PMC5610412; doi:10.1186/s12936-017-2029-z)

A

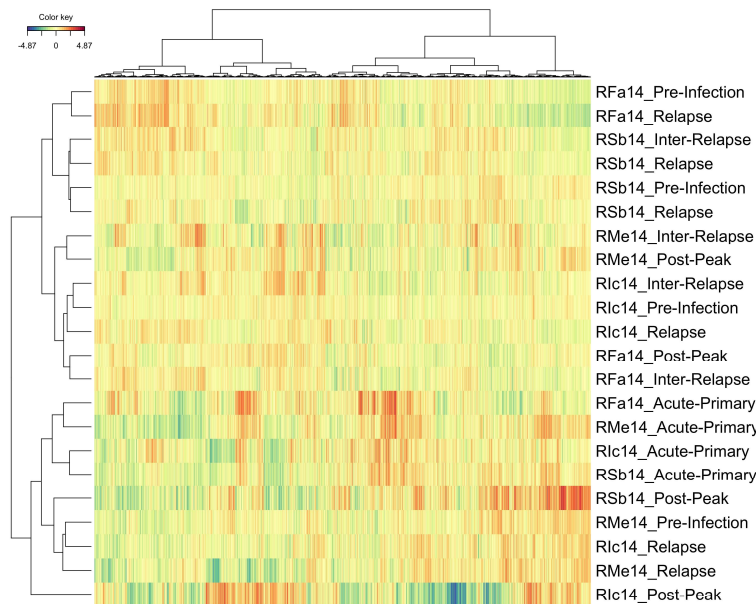

B

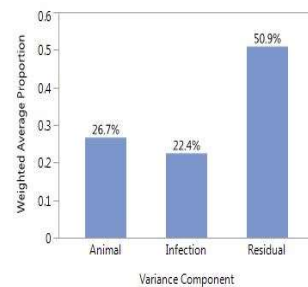

C

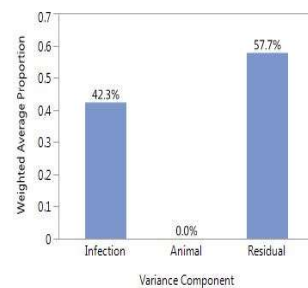

Supplement: Supplementary file 1 — Additional file 1. Overview of the BM transcriptome prior to SNM transformation. (A) Clustered heatmap of the BM transcriptome before animal effects were removed. Four samples of acute primary infection form a cluster separated from other infection points. Other infection points do not form unique or separated clusters, with many of the most closely related samples coming from the same animals, suggesting that individual effects may confound the ability to identify the effect of infection points on gene expression profiles. Colours indicate z-score normalized expression values. (B) Variance Component Analysis showing that individual animal effects originally explain 26.7% of the gene expression variance, while infection points explain 22.4% of the gene expression variance. (C) After removing individual effects by SNM, infection points explain 42.3% of the gene expression variance. [file 12936_2017_2029_MOESM1_ESM.pdf]

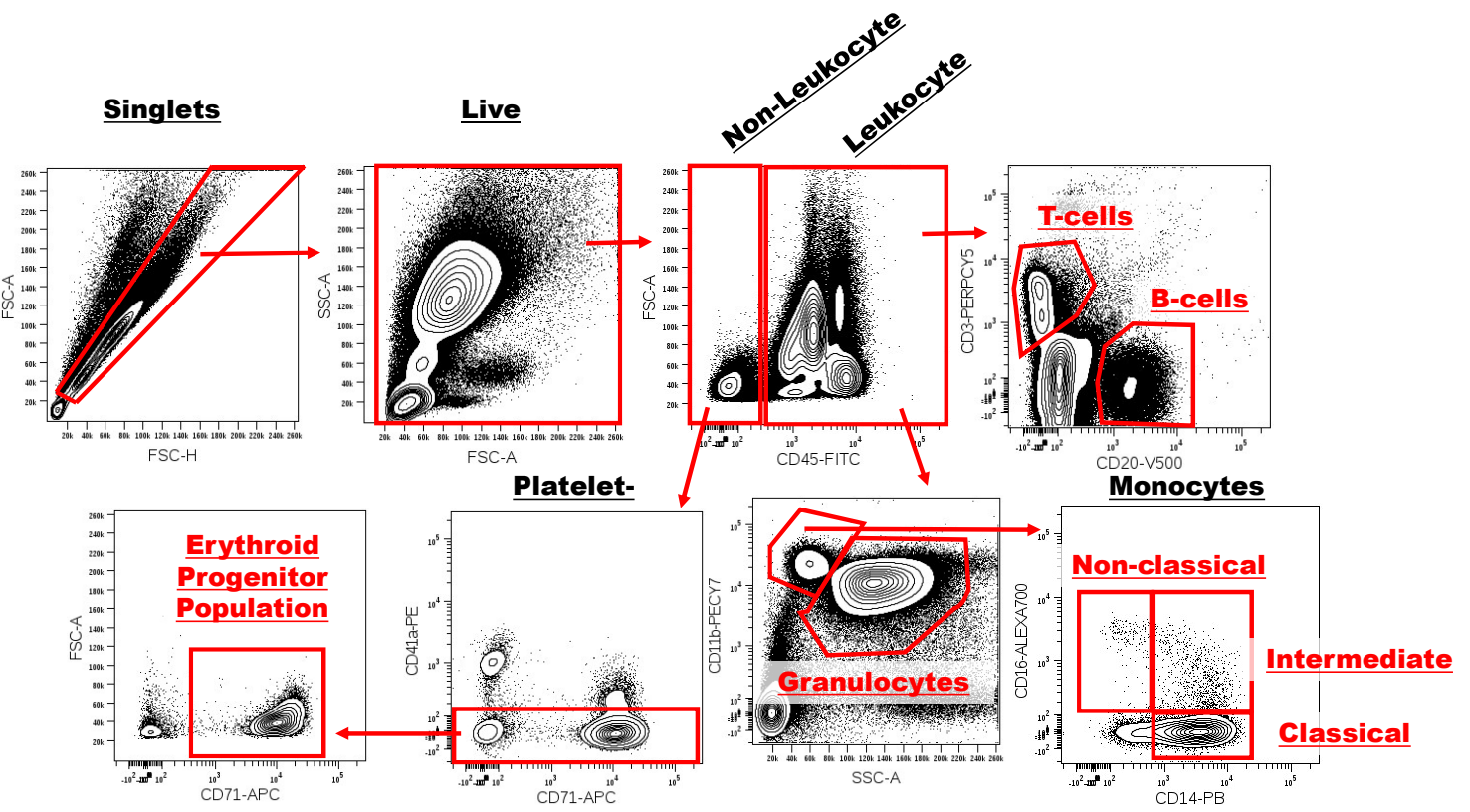

Supplement: Supplementary file 7 — Additional file 7. Flow cytometry gating strategy to monitor cellular subset in the bone marrow of rhesus macaques with malaria. A representative gating strategy for determining the frequency of various immune cell subsets in bone marrow aspirate collected from rhesus macaques during P. cynomolgi infection. This representative plot is from a pre-infection time-point. [file 12936_2017_2029_MOESM7_ESM.pdf]

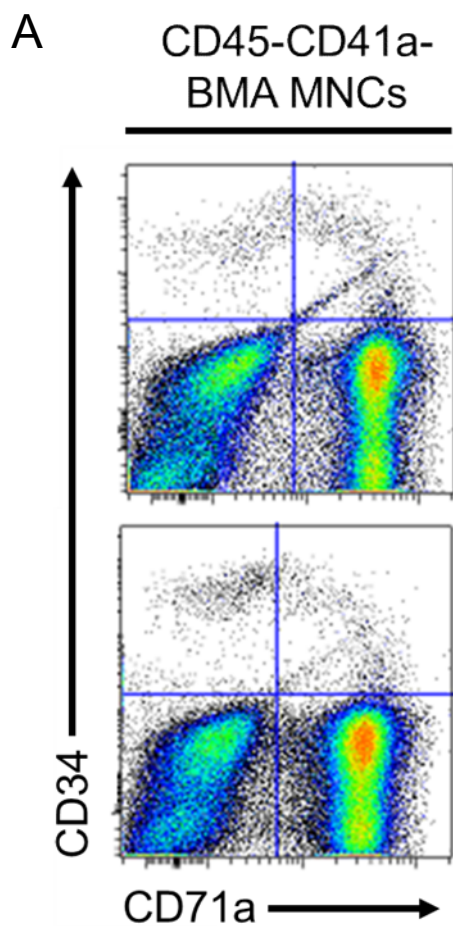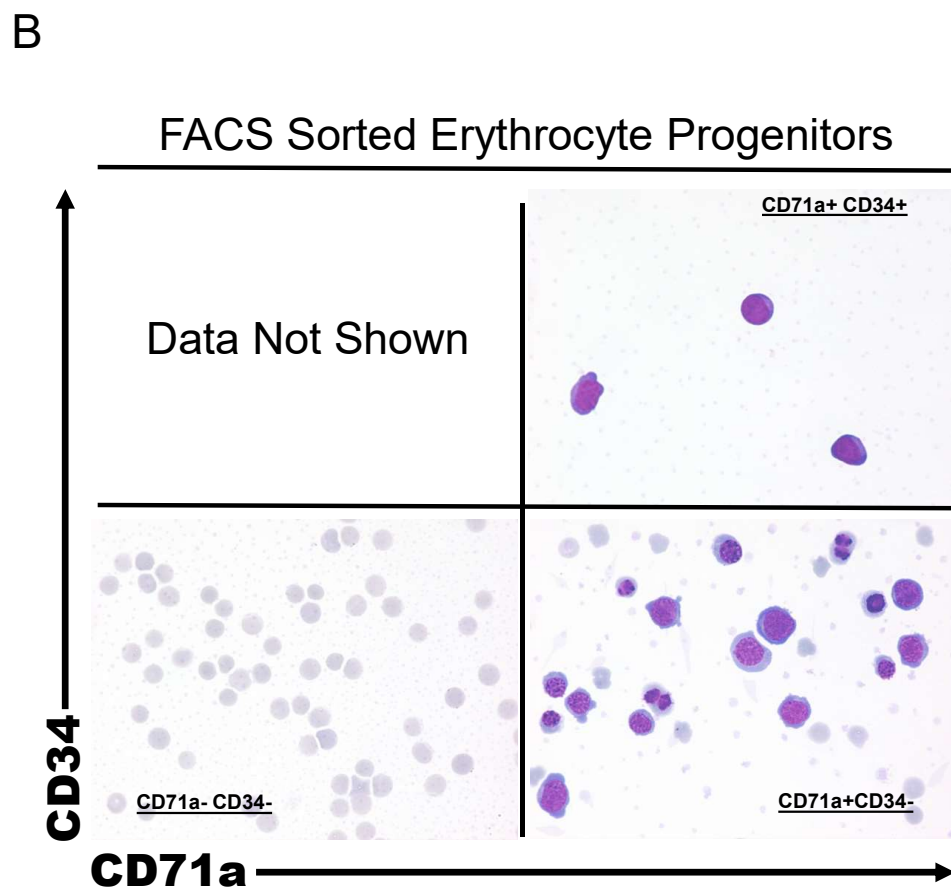

Supplement: Supplementary file 8 — Additional file 8. Characterization of the cellular subsets constituting the erythroid progenitor cell population. (A) FACS profiles corresponding to (B). (B) Cells consistent with the cell surface phenotype of erythroid progenitors were identified based on CD34 and CD71a surface staining. [file 12936_2017_2029_MOESM8_ESM.pdf]

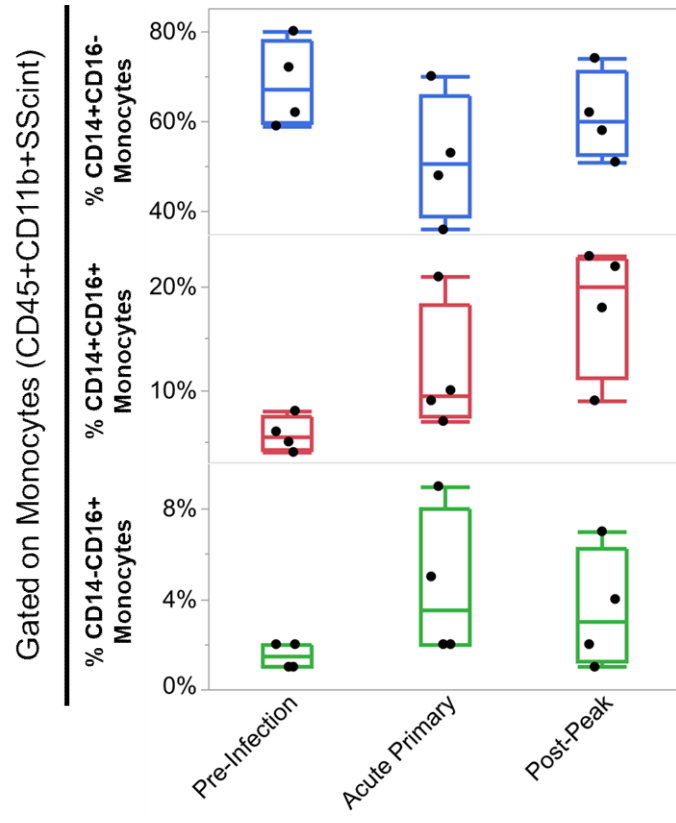

Supplement: Supplementary file 9 — Additional file 9. Changes in monocyte subsets in the bone marrow during an initial blood-stage P. cynomolgi infection. The percentage of classical (CD14+CD16−), intermediate (CD14+CD16+), and non-classical (CD14−CD16+) monocytes out of the monocyte compartment are shown before infection, during the acute primary infection, and after the peak of infection. [file 12936_2017_2029_MOESM9_ESM.pdf]

# Differentially Expressed Genes in EPO Pathway

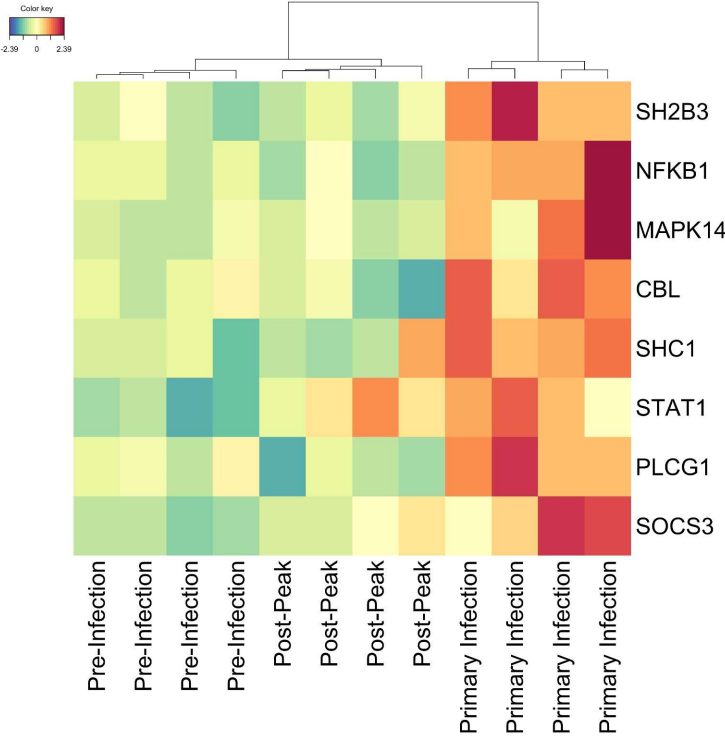

Supplement: Supplementary file 10 — Additional file 10. Transcriptional profiles of genes in the EPO pathway before, during, and immediately after acute malaria. The genes in the erythropoietin pathway identified as differentially expressed in the bone marrow RNA-Seq data set are shown. Samples are hierarchically clustered. Colours indicate z-score normalized expression values. [file 12936_2017_2029_MOESM10_ESM.pdf]
